# PP09 Fecal Immunochemical Tests For Patients With Symptoms Suggestive Of Colorectal Cancer: A Systematic Review And Multiple-Threshold Meta-Analysis

**DOI:** 10.1017/S026646232400182X

**Published:** 2025-01-07

**Authors:** Sue Harnan, Jean Hamilton, Emma Simpson, Mark Clowes, Aline Navega Biz, Sophie Whyte, Shijie Ren, Katy Cooper, Alex Ball, Sally Benton, Rachel Carten, Matt Kurien, Kevin Monahan, Laura Heathcote, Matt Stevenson

## Abstract

**Introduction:**

Extending fecal immunochemical tests for hemoglobin (FITs) to primary care patients with high-risk symptoms suggestive of colorectal cancer (CRC) could reduce colonoscopy waiting lists, enabling earlier treatment. Higher FIT thresholds could decrease referrals but increase missed disease compared with lower thresholds. We aimed to systematically review and synthesize test accuracy data across thresholds for use in a cost-effectiveness analysis.

**Methods:**

Searches across ten sources were conducted (December 2022). Included were diagnostic accuracy studies of HM-JACKarc, OC-Sensor, FOB Gold, QuikRead go, NS-Prime, and four Immunodiagnostik (IDK) tests in patients presenting to, or referred from, primary care with symptoms suggestive of CRC using any reference standard. Risk of bias was assessed with QUADAS-2. Syntheses of sensitivity and specificity at all reported thresholds were planned for each test to provide summary estimates at all possible thresholds within the observed range. Sensitivity analyses investigating population type and reference standard, and subgroup analyses by patient characteristics (e.g., anemia, age, sex, ethnicity) were conducted.

**Results:**

HM-JACKarc (n=16 studies) sensitivity ranged from 95.9 percent (95 percent credible interval [95% CrI]: 92.7, 97.9) to 46.3 percent (95% CrI: 37.4, 54.9) and specificity from 65.1 percent (95% CrI: 55.6, 74.8) to 97.7 percent (95% CrI: 94.7, 99.2) (thresholds 2 and 400 μg hemoglobin/g feces [μg/g], respectively). OC-Sensor (n=11) sensitivity ranged from 94.2 percent (95% CrI: 91.2, 96.7) to 54.2 percent (95% CrI: 48.4, 60.2) and specificity from 62.7 percent (95% CrI: 47.4, 77.2) to 97.3 percent (95% CrI: 92.9, 99.3) (thresholds 4 and 200 μg/g, respectively). FOB Gold (n=3) sensitivity ranged from 91.4 percent (95% CrI: 71.6, 99.6) to 73.9 percent (95% CrI: 53.8, 91.2) and specificity from 78.1 percent (95% CrI: 70.0, 86.0) to 96.4 percent (95% CrI: 92.6, 98.9) (thresholds 2 and 150 μg/g, respectively). There were limited or no data on the other tests.

**Conclusions:**

Sensitivity and specificity were synthesized for three tests only, since data for the remaining tests were extremely limited or absent. Even at the lowest threshold, none of the tests had perfect sensitivity. Future studies should further investigate comparative accuracy and the impact of patient characteristics, patient recruitment criteria, and the reference standard on estimates of diagnostic test accuracy.

